# Diaphragm thickening fraction predicts noninvasive ventilation outcome: a preliminary physiological study

**DOI:** 10.1186/s13054-021-03638-x

**Published:** 2021-06-26

**Authors:** Giovanna Mercurio, Sonia D’Arrigo, Rossana Moroni, Domenico Luca Grieco, Luca Salvatore Menga, Anna Romano, Maria Giuseppina Annetta, Maria Grazia Bocci, Davide Eleuteri, Giuseppe Bello, Luca Montini, Mariano Alberto Pennisi, Giorgio Conti, Massimo Antonelli

**Affiliations:** 1 Department of Anesthesiology, Intensive Care and Emergency Medicine, Fondazione Policlinico Universitario A. Gemelli IRCCS, Largo A. Gemelli, 8, 00168 Rome, Italy; 2 Biostatistics, Office of the Scientific Director, Fondazione Policlinico Universitario A. Gemelli IRCCS, Rome, Italy; 3 Institute of Anesthesiology and Intensive Care Medicine, Catholic University of the Sacred Heart, Rome, Italy

**Keywords:** Acute respiratory failure, Noninvasive ventilation, Ultrasound, Diaphragm thickening fraction, Rapid shallow breathing index

## Abstract

**Background:**

A correlation between unsuccessful noninvasive ventilation (NIV) and poor outcome has been suggested in de-novo Acute Respiratory Failure (ARF) patients. Consequently, it is of paramount importance to identify accurate predictors of NIV outcome. The aim of our preliminary study is to evaluate the Diaphragmatic Thickening Fraction (DTF) and the respiratory rate/DTF ratio as predictors of NIV outcome in de-novo ARF patients.

**Methods:**

Over 36 months, we studied patients admitted to the emergency department with a diagnosis of de-novo ARF and requiring NIV treatment. DTF and respiratory rate/DTF ratio were measured by 2 trained operators at baseline, at 1, 4, 12, 24, 48, 72 and 96 h of NIV treatment and/or until NIV discontinuation or intubation. Receiver operating characteristic (ROC) curve analysis was performed to evaluate the ability of DTF and respiratory rate/DTF ratio to distinguish between patients who were successfully weaned and those who failed.

**Results:**

Eighteen patients were included. We found overall good repeatability of DTF assessment, with Intra-class Correlation Coefficient (ICC) of 0.82 (95% confidence interval 0.72–0.88). The cut-off values of DTF for prediction of NIV failure were < 36.3% and < 37.1% for the operator 1 and 2 (*p* < 0.0001), respectively. The cut-off value of respiratory rate/DTF ratio for prediction of NIV failure was > 0.6 for both operators (*p* < 0.0001).

**Conclusion:**

DTF and respiratory rate/DTF ratio may both represent valid, feasible and noninvasive tools to predict NIV outcome in patients with *de-novo* ARF.

*Trial registration* ClinicalTrials.gov Identifier: NCT02976233, registered 26 November 2016.

## Background

Over the last 2 decades, it has been suggested that noninvasive ventilation (NIV) might be effective in avoiding intubation [1, 2] and improving survival in the acute care setting [3] when compared to conventional oxygen therapy [4].

Nonetheless, its use in de-novo Acute Respiratory Failure (ARF) is debated, since a strict association between the unsuccessful NIV and the poor outcome has been suggested [5]. Given the risks associated with either premature or delayed NIV discontinuation [6], evaluating weaning readiness, as well as correct timing of intubation, is a critical challenge in patients with de-novo ARF.

Up to now, there are few available bedside measurements for prediction of NIV outcome and the decision to discontinue NIV is mainly based on clinical and physiologic parameters [7, 8].

Indeed, although sophisticated methods have been proposed to predict weaning outcome [8], none of these methods has ever achieved a wide bedside use because of their invasiveness, of the inconsistent results, and of the need for trainee personnel and complicated equipment. Consequently, the rapid shallow breathing index is still preferable to these methods because of its simpler application and more immediate interpretation [9].

The diaphragm is the main respiratory muscle, and its dysfunction has been associated with prolonged mechanical ventilation and weaning failure [10, 11].

Recently, ultrasound has been used at the bedside in acutely ill patients both for rapid functional assessment of the skeletal muscles [12] and for evaluation of diaphragmatic function [13] with minimal invasiveness and without any X-ray exposure. Ultrasound can be used to measure the excursions of the diaphragm, its thickness and its speed of contraction [14, 15], yielding information about the muscle function and the respiratory efficiency.

Indeed, diaphragmatic thickness (DT) correlates with the strength and the shortening of the muscle [16, 17]. The volume of muscle mass remains constant during diaphragm contraction. Consequently, as the muscle shortens it becomes thicker, so that DT changes are inversely related to changes in the length of the muscle. Moreover, the magnitude of diaphragm shortening and contraction may predict successful extubation in invasively ventilated patients [18].

Recently, Vivier et al. [15] have conducted a physiological study to assess the accuracy of Diaphragm Thickening Fraction (DTF) and its contribution to the respiratory workload in 12 critically ill patients requiring planned NIV after extubation. The patients were studied either while spontaneously breathing or during NIV at three different levels of pressure support, measuring DTF and diaphragmatic Pressure–Time Product per breath (PTP_di_).

These authors found that increases in the level of Pressure Support were associated with a reduction in both PTP_di_ and DTF, and that there was a significant correlation between PTP_di_ and DTF (*ρ* = 0.74, *p* < 0.001). They concluded that DTF is a noninvasive method that may be useful in evaluating the diaphragm contribution to the respiratory workload in acutely ill patients undergoing NIV treatment.

The aim of this preliminary study is to assess whether measurements of DTF and its relationship with respiratory rate may predict NIV outcome in patients with de-novo ARF admitted to the emergency department.

## Methods

All patients with de-novo ARF requiring NIV treatment admitted to the emergency department at Fondazione Policlinico Universitario A. Gemelli, IRCCS Rome-Italy were included into this preliminary study.

The protocol was approved by the local Ethics Committee (Prot 20813/16 ID 1200) and informed consent was obtained by all study participants.

The criteria for eligibility were de-novo ARF in the presence of respiratory rate ≥ 35 breaths per minute, a ratio of the PaO_2_ to the fraction of inspired oxygen (PaO_2_/FiO_2_) of less than 200 with oxygen therapy through a Venturi mask or a High Flow Nasal Cannula (50 L/min) and active contraction of the accessory muscles of respiration or paradoxical abdominal motion.

The exclusion criteria were: age < 18 years, pregnancy, diaphragm paralysis, exacerbation of asthma and/or chronic obstructive pulmonary disease, neuromuscular disorders, severe obesity with Body Mass Index (BMI) ≥ 35 kg/m^2^, palliative NIV in patients with malignancy, ineffective cough and/or inability to protect airways.

Noninvasive Pressure Support Ventilation (PSV) with positive end-expiratory pressure (PEEP) was delivered through either a facial mask with an inflatable soft cushion seal (Gibeck, Upplands, Sweden; Vitalsigns, Towota, NJ, USA) or with a clear, latex-free helmet (CaStar, Starmed, Mirandola, Italy), according to a clinical decision.

In the patients with a facial mask, PSV was started at 10 cmH_2_O and increased with progressive stepwise increments of 2–3 cmH_2_O, to obtain an exhaled tidal volume of 6 mL/kg, respiratory rate ≤ 25 breaths per minute, patient comfort and disappearance of accessory muscle activity or paradoxical abdominal motion. PEEP was increased with stepwise increments of 2–3 cmH_2_O up to 12 cmH_2_O to ensure peripheral oxygen saturation (SpO_2_) of ≥ 90% with the lowest possible FiO_2_.

In the patients with a helmet, a soft cushion around the neck provides sealing of the interface, reducing air leaks and allowing high levels of PEEP. PEEP was set to 10–12 cmH_2_O to ensure adequate inflation of the interface, and since part of the volume delivered to the system was used to distend the helmet without reaching the patient PS levels were increased with stepwise increments of 2–3 cmH_2_O up to 15–18 cmH_2_O. Ventilator settings were then adjusted according to SpO_2_ and measurements of arterial blood gases. The flow trigger was set at 3 L/min, checking out for the absence of auto-triggering phenomena.

### Criteria for immediate intubation

Criteria for immediate intubation included the inability to maintain a PaO_2_/FiO_2_ > 140, the onset of seizures or coma (Glasgow coma score ≤ 8), hemodynamic instability (systolic blood pressure ≤ 80 mmHg despite adequate fluid resuscitation and/or increased needs of vasopressors [norepinephrine > 0.5 mcg/kg/min] and/or electrocardiographic signs of ischemia or arrhythmias), intolerance of the interface, ineffective cough, discomfort, or the need for an urgent surgery. After intubation, all patients were ventilated with a low-tidal-volume protective ventilatory strategy [19].

### Criteria for NIV weaning

NIV support was progressively reduced in accordance with the degree of both gas exchange and clinical improvement and discontinued when the patient was able to maintain respiratory rate ≤ than 25 breaths per minute and PaO_2_ > 75 mmHg with a FiO_2_ of 0.5 without ventilatory support.

### Definitions and measurements

On the emergency department admission, we recorded the patients’ characteristics, including the Simplified Acute Physiologic Score (SAPS II) [20], the Richmond Agitation-Sedation Scale (RASS) [21], the diagnosis and the comorbidities.

Arterial blood gas levels, respiratory and hemodynamic parameters were measured at baseline, at 1, 4, 12, 24, 48, 72 and 96 h or until intubation.

Improvement in gas exchange was defined as the ability to increase PaO_2_/FiO_2_ above 200 or to increase this ratio > 100 over the baseline.

Improvement in respiratory rate was defined as a respiratory rate ≤ 25 breaths per minute or disappearance of accessory muscles’ use and paradoxical abdominal motion.

NIV success was defined as the improvement in respiratory rate and gas exchange within the first 96 h of treatment.

The need of intubation and/or failure to reach an improvement in respiratory rate and gas exchange at any point of the study was defined NIV failure.

Patients were monitored for the development of sepsis, septic shock and Acute Respiratory Distress Syndrome (ARDS) [22, 23].

We also recorded the duration of mechanical ventilation, the emergency department and the hospital length of stay, as well as the emergency department and in-hospital mortality.

### Sonographic definitions and measurements

All patients were placed in a semi-recumbent position with the right arm elevated. DT was assessed through a linear high-frequency (7–10 MHz) probe (Vivid E, General Electrics). The right diaphragm was imaged by placing the probe perpendicular to the chest wall on the mid-axillary line at the apposition point of the diaphragm, between the 8th and the 10th intercostal spaces [15]. In this area, the diaphragm is visualized as a three layer structure with a non-echogenic central layer between two echogenic layers, the peritoneum and the diaphragmatic pleurae. The change in DT between end-expiration and end-inspiration (DTF) was expressed as (DT_end-inspiration_ − DT_end-expiration_/DT_end-expiration_) × 100 [15]. In each patient, DTF was estimated as the mean value measured in three to five breaths and the examinations were carried out in blind by 2 different and appropriately trained operators [24].

We calculated the ratio between respiratory rate and DTF (respiratory rate/DTF) at any time point.

Images were obtained at baseline, at 1, 4, 12, 24, 48, 72 and 96 h of NIV treatment and/or until NIV discontinuation or intubation.

### Objectives

The primary objective of our study was to assess the feasibility of ultrasound measurement of DTF and its accuracy in predicting NIV outcome in patients with de-novo ARF.

We also aimed to evaluate the possibility of predicting the need of endotracheal intubation using DTF and its relationship with respiratory rate.

### Statistics

Statistical analysis (MedCalc Software bvba, Ostend, Belgium; http://www.medcalc.org; 2014) was preliminarily performed on 18 patients. This sample size was esteemed to be appropriate, since it was enough to identify a disagreement rate between operators of *π*_D_ = 5%, with *α* = 0.05 and power = 80%, with regards to the primary study objectives.

Continuous variables with normal distributions were expressed as means and standard deviation (SD) and assessed with the Student’s t-test while those with non-normal distributions were expressed as medians and interquartile ranges [IQR] and assessed with the Mann–Whitney test.

Repeated measures over time were evaluated with the analysis of variance (ANOVA).

Normality of data was verified with the Kolmogorov–Smirnov test. Categorical variables were presented as group proportions and analyzed with the Chi-square test or Fisher exact’s test, as appropriate. A two-tailed *p* value of less than 0.05 was considered statistically significant.

Receiver operating characteristic (ROC) curve analyses were performed to assess the ability of both DTF and respiratory rate/DTF ratio to distinguish between patients who succeeded weaning and those who failed it. The optimal criterion value (cut-off value) took into account sensitivity and specificity.

As regarding the ultrasound measurements, the reproducibility was expressed by the ICC [25].

## Results

**Fig. 1:**
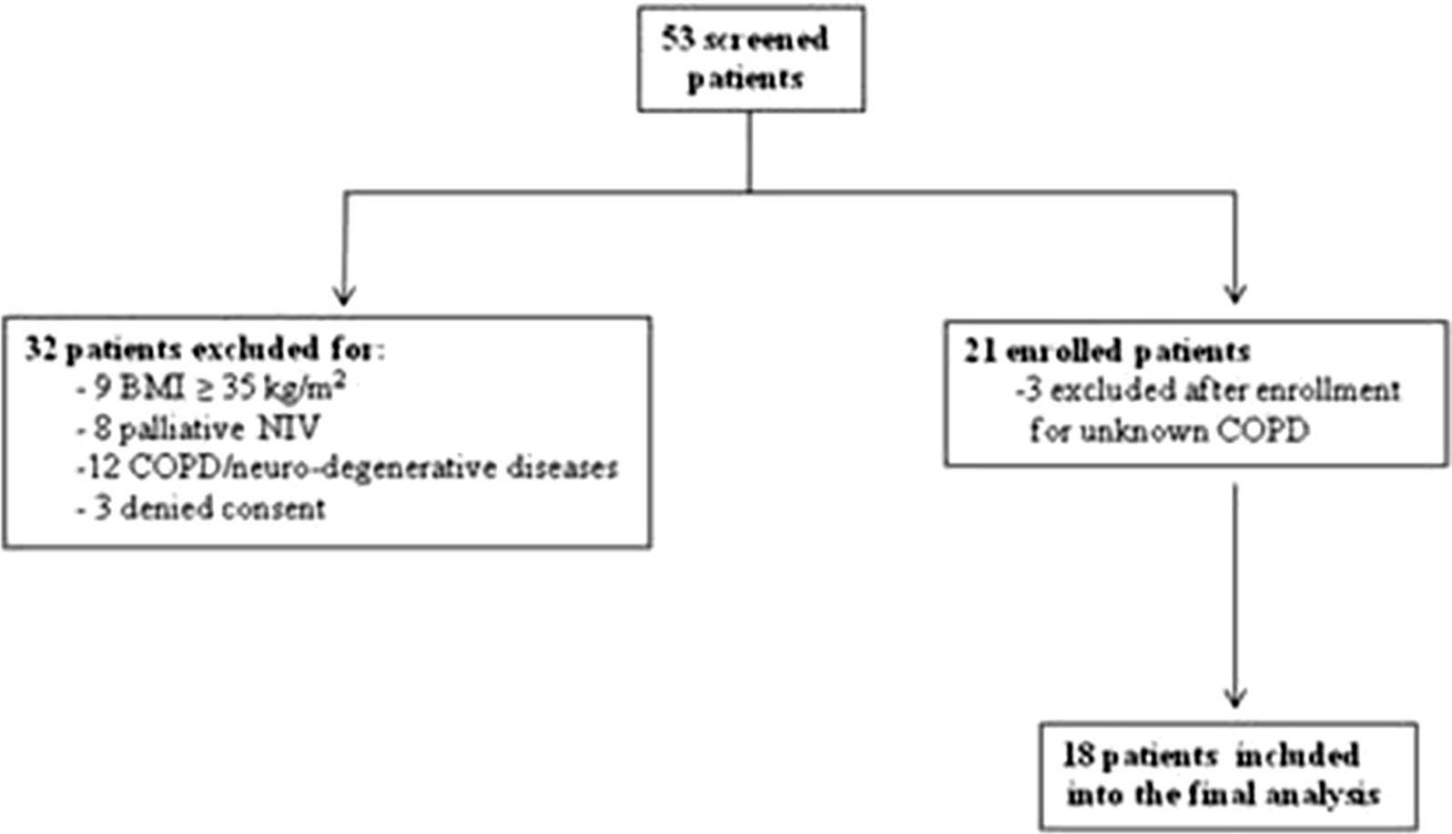
Flow chart of screened and enrolled patients

Over a period of 36 months, 53 eligible patients were screened for inclusion. Of these, 32 patients were excluded and 21 were enrolled. Three patients were excluded after inclusion for a concomitant diagnosis of chronic obstructive pulmonary disease unknown at the time of enrolment, so that only18 patients (8 males, 44.5%) were included into the final analysis (Fig. 1).

Mean age (SD) was 66 (19) years, with a mean (SD) SAPS II score of 44 (4). On admission to the emergency department the median [IQR] PaO_2_/FiO_2_ ratio was 89.4 [77.4–117.5], the median PaCO_2_ was 30.6 [29.3–39.2] mmHg, and the median respiratory rate was 38 [36–44.2] breaths per minute. The cause of acute hypoxic respiratory failure on admission was pneumonia in more than 70% of included patients.

Demographic and clinical characteristics of the population are reported in Table 1.

**Table 1:** Characteristics of patients at baseline, in the whole population

Characteristics	All patients (*n* = 18)
Age (years)	66 ± 19
Male, *n* (%)	8 (44.5)
^a^BMI (kg/m^2^)	25 ± 4
^b^SAPS II score	44 ± 4
RASS score	1 [0–1]
PaO_2_/FiO_2_ (mmHg)	89.4 [77.4–117.5]
PaCO_2_ (mmHg)	30.6 [29.3–39.2]
Respiratory rate, breaths per minute	38 [36–44.2]
Acute hypoxemic respiratory failure causes on emergency department admission	
Pneumoniae, *n* (%)	13 (72.2)
Post-surgery, *n* (%)	1 (5.5)
Abdominal abscess, *n* (%)	1 (5.5)
Hepato-renal syndrome, *n* (%)	1 (5.5)
Non cardiogenic pulmonary oedema, *n* (%)	2 (11.1)
Comorbidities	
Cardiovascular diseases, *n* (%)	10 (55.5)
Renal diseases, *n* (%)	5 (27.7)
Lung diseases, *n* (%)	2 (11.1)
Diabetes (%)	4 (22.2)
Oncologic diseases (%)	2 (11.1)
Immunocompromised state (%)	1 (5.5)
Outcome	
NIV failure, *n* (%)	10 (55.5)
Time to intubation (h)	12.5 [9.5–24.2]
Length of stay in emergency department (days)	6 [3.2–9]
Hospital length of stay (days)	17 [12–29]
Emergency department mortality, *n* (%)	6 (33)
In-hospital mortality, *n* (%)	8 (44.4)

Values are displayed as means ± SD or as medians [interquartile range] when appropriate. Normal distribution was assessed with the Kolmogorov–Smirnov test

FiO_2_ denotes fraction of inspired oxygen,

PaCO_2_ partial pressure of arterial carbon dioxide, and PaO_2_ partial pressure of arterial oxygen

^a^The body-mass index is the weight in kilograms divided by the square of the height in meters

^b^The Simplified Acute Physiology Score (SAPS) II was calculated from 17 variables at enrolment, information about previous health status and information obtained at admission. Scores range from 0 to 163, with higher scores indicating more severe disease

### Sonographic measurements

We found overall good repeatability of DTF assessment, with an ICC of 0.82 (95% CI 0.72–0.88).

**Fig. 2:**
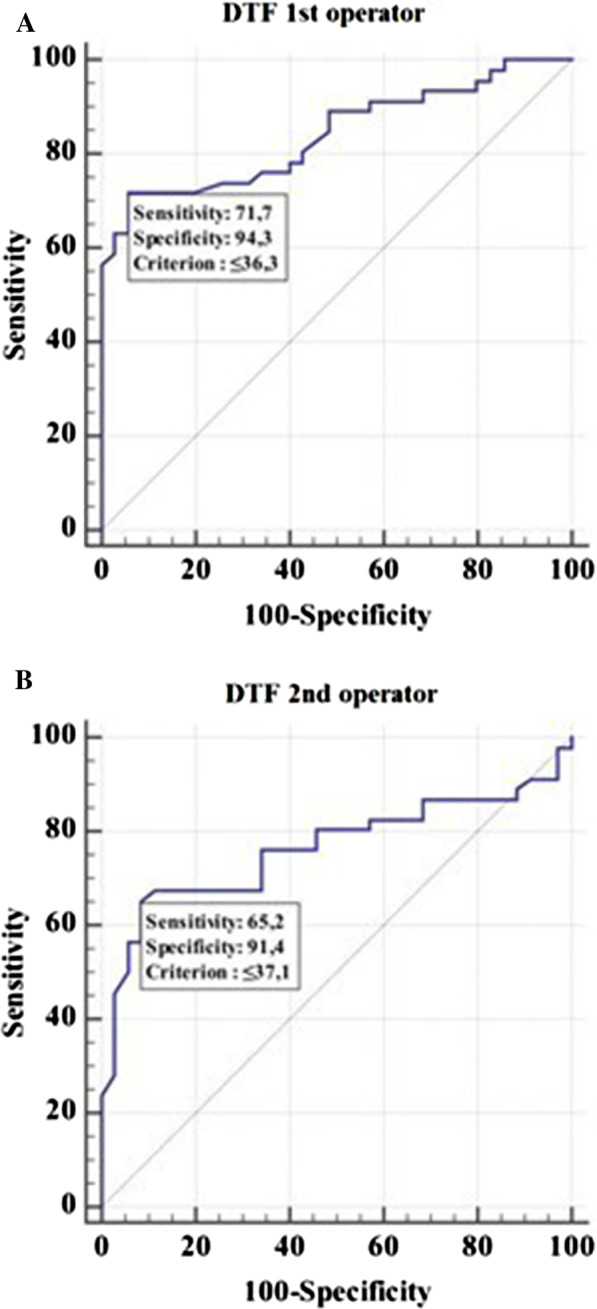
Receiver operator characteristic curve (ROC) to assess the ability of the Diaphragm Thickening Fraction (DTF) to predict noninvasive ventilation (NIV) outcome for operator 1 (**A**) and 2 (**B**), respectively. Area under the ROC curve (AUC) 0.84 (95% CI 0.74 to 0.91) for operator 1 and 0.76 (95% CI 0.65 to 0.85) for operator 2

The cut-off value of DTF < 36.3% for the operator 1 significantly predicted NIV failure (*p* < 0.0001) with sensitivity of 71.7% (95% CI 56.5–84.0) and specificity of 94.3% (95% CI 80.8–99.3) (Fig. 2A).

The cut off value of DTF < 37.1% for the operator 2 significantly predicted NIV failure (*p* < 0.0001) with sensitivity of 65.2% (95% CI 49.8–78.6) and specificity of 91.4% (95% CI 76.9–98.2) (Fig. 2B).

**Fig. 3:**
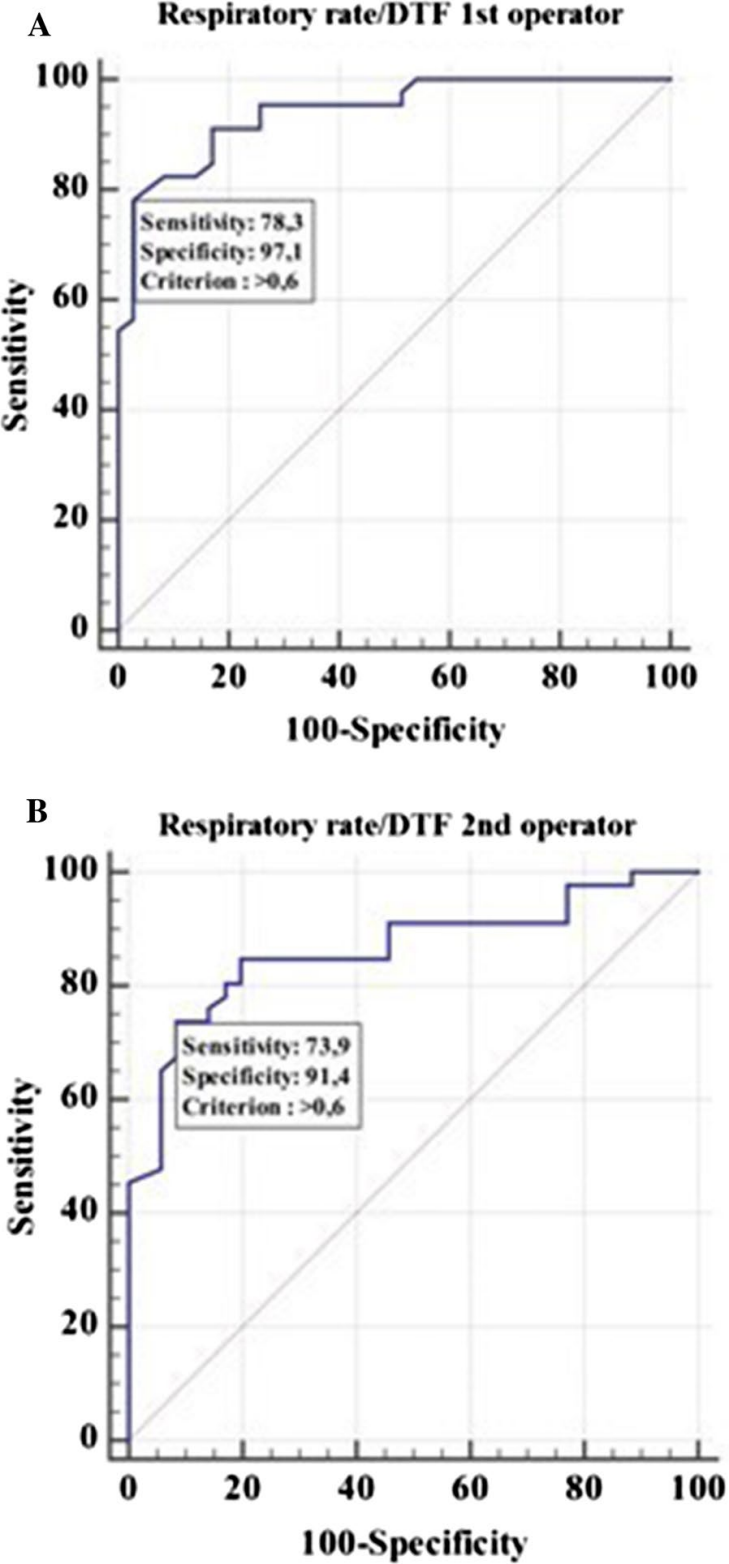
Receiver operator characteristic curve (ROC) to assess the ability of the respiratory rate/DTF to predict noninvasive (NIV) outcome for operator 1 (**A**) and 2 (**B**), respectively. Area under the ROC curve (AUC) 0.94 (95% CI 0.86 to 0.98) for operator 1 and 0.86 (95% CI 0.77 to 0.93) for operator 2

We also found that the cut-off value of respiratory rate/DTF that distinguished between NIV failure and success was > 0.6 for the operator 1 (*p* < 0.0001) with sensitivity of 78.3% (95% CI 63.6–89.1) and specificity of 97.1% (95% CI 85.1–99.9) (Fig. 3A).

The cut-off value of respiratory rate/DTF that distinguished between NIV failure and success was also > 0.6 for the operator 2 (*p* < 0.0001) with sensitivity of 73.9% (95% CI 58.9–85.7) and specificity of 91.4% (95% CI 76.9–98.2) (Fig. 3B).

**Fig. 4:**
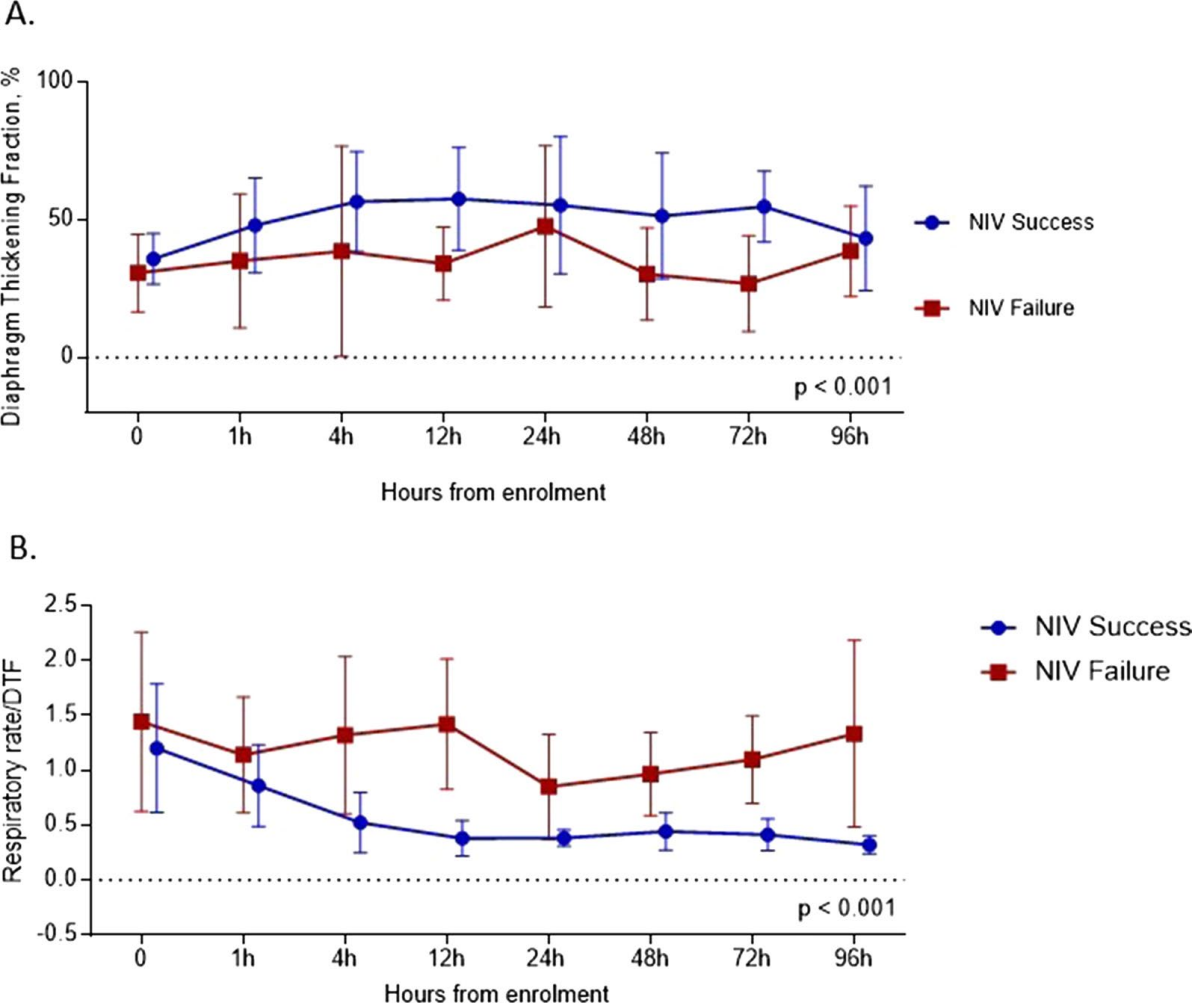
DTF (**A**) and respiratory rate/DTF (**B**) in the NIV success and in the NIV failure group over time; the values displayed are the mean of the measures of the two operators. Patients were censored after endotracheal intubation. Comparisons between groups were performed with 1-way analysis of variance. Each point on the graph represents the mean values, and error bars represent Standard Deviation

NIV success patients compared to NIV failure patients had significantly higher mean (SD) DTF in the first 96 h [52 (23) % vs 34 (19) %, *p* < 0.001] (Fig. 4A) and lower respiratory rate/DTF [0.58 (0.39) vs 1.24 (0.63), *p* < 0.001] (Fig. 4B).

### Clinical outcomes

NIV median time [IQR] without discontinuation was 3 [2.2–32.2] hours for patients that did not need endotracheal intubation, and 12.5 [9.5–24.2] hours for the ones that required endotracheal intubation.

The rate of NIV failure was 55.5% (10 out of 18). Patients who required intubation among these were 80% (8 out of 10).

**Fig. 5:**
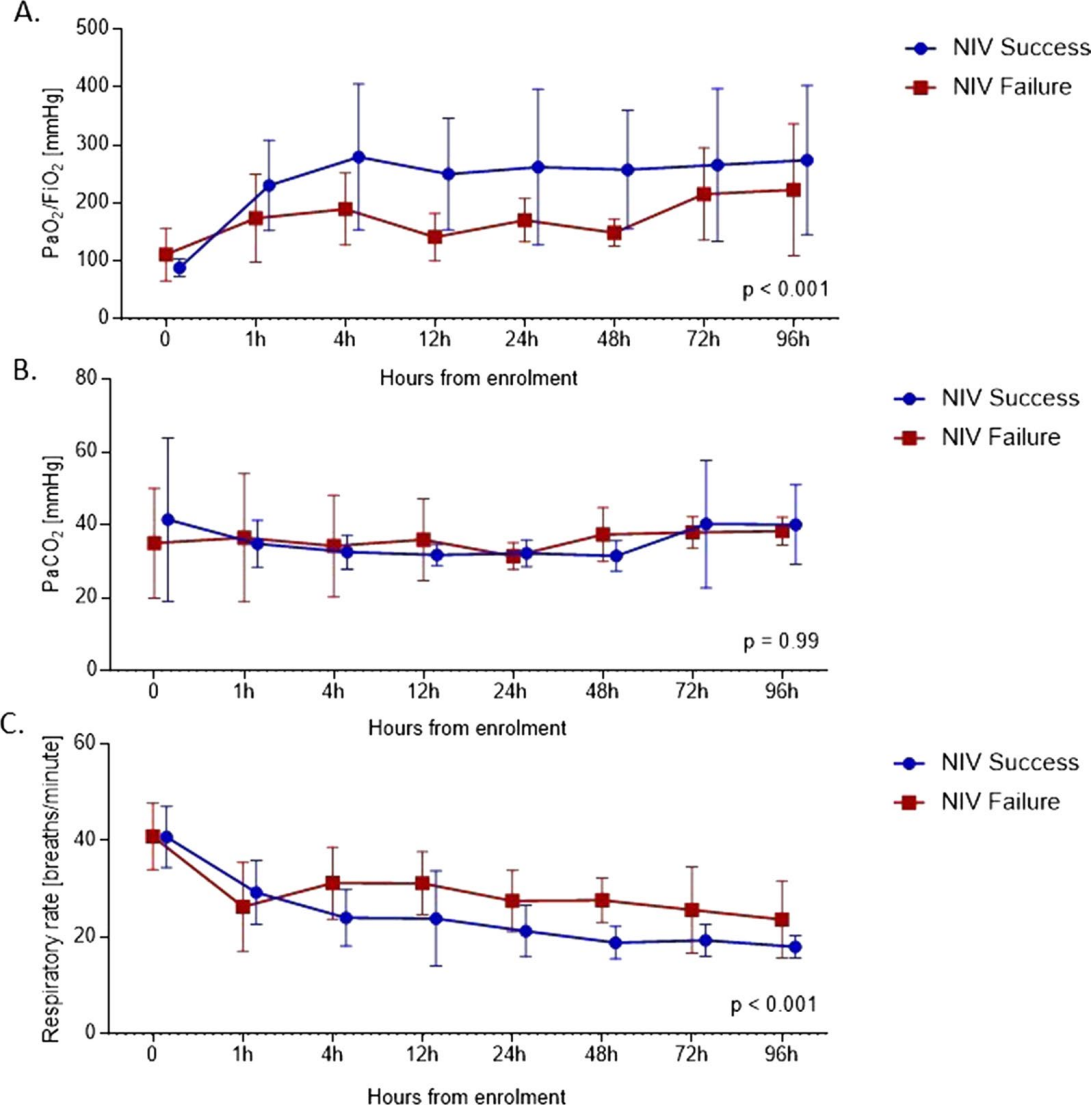
PaO_2_ (**A**), PaCO_2_ (**B**) and respiratory rate (**C**) in the NIV success and in the NIV failure group over time. Patients were censored after endotracheal intubation. Comparisons between groups were performed with 1-way analysis of variance. Each point on the graph represents the mean values, and error bars represent Standard Deviation

NIV success patients compared to NIV failure patients had significantly higher mean (SD) PaO_2_/FiO_2_ in the first 96 h [236 (118) vs 163 (67), *p* < 0.001], lower respiratory rate [24 (9) vs 30 (9) breaths per minute, *p* < 0.001] and similar PaCO_2_ [35 (11) vs 35 (13) mmHg, *p* = 0.99] (Fig. 5A–C, Table 2).

**Table 2:** Physiological outcomes in the two groups in the first 96 h

Outcome	Study group		*P* value
	NIV success (*n* = 8)	NIV failure (*n* = 10)	
Diaphragm thickening fraction in the first 96 h after enrolment (%)			
1st operator	54.6 (47.3–61.9)	31.8 (26.1–37.6)	< 0.001
2nd operator	48.1 (42.9–53.3)	37.3 (29.8–44.8)	0.016
Mean between the 2 operators	52.1 (45.7–58.5)	34.3 (28.6–40)	< 0.001
Respiratory rate/Diaphragm thickening fraction in the first 96 h after enrolment			
1st operator	0.55 (0.45–0.66)	1.4 (1.1–1.6)	< 0.001
2nd operator	0.61 (0.49–0.73)	1.1 (0.89–1.28)	< 0.001
Mean between the 2 operators	0.58 (0.47–0.69)	1.24 (1.05–1.42)	< 0.001
PaO_2_/FiO_2_ ratio in the first 96 h after enrolment (mmHg)			
	236 (206–267)	163 (144–183)	< 0.001
PaCO_2_ in the first 96 h after enrolment (mmHg)			
	35 (32–38)	35 (32–39)	0.99
Respiratory rate in the first 96 h after enrolment, breaths per minute			
	24 (22–27)	31 (28–33)	< 0.001

Results are displayed as Means (95% CI). *P* values are calculated with one-way ANOVA

*NIV* non-invasive ventilation

During stay in the emergency department, ARDS was described in 6 patients (33%), sepsis in 1 patient (5%) and septic shock in 4 patients (22%). The median [IQR] length of stay in the emergency department and in-hospital were 6 [3.2–9] and 17 [12–29] days, respectively.

The overall mortality in the emergency department was 33.3% and it was higher in intubated than in non-intubated patients (75% vs 0%, *p* = 0.023).

The means (95%CI) of DTF (%) and the respiratory rate/DTF obtained by two operators between NIV success and NIV failure during the first 96 h are reported in Table 3.

**Table 3:** Physiological outcomes in the two groups at different study time points

Outcome	Study group		*P* value
	NIV success (*n* = 8)	NIV failure (*n* = 10)	
Diaphragm thickening fraction (%)			
At the enrolment	35 [25.8–67.4]	41.5 [22–56]	0.864
1 h after the enrolment	37.9 [25.2–50.9]	30.1 [17.8–43.6]	0.41
4 h after the enrolment	54.2 [41–72.6]	28.2 [19.2–53.8]	0.114
12 h after the enrolment	53 [39.3–74]	27.4 [21.9–34.5]	0.032
24 h after the enrolment	55.1 [38–80.9]	33.3 [26.9–63.9]	0.114
48 h after the enrolment	43.3 [39–54.4]	32.5 [23.8–32.5]	0.067
72 h after the enrolment	47.5 [39–56]	19.7 [15.1–19.7]	0.383
96 h after the enrolment	56.9 [45.7–80.3]	23.3 [15.8–23.3]	0.071
Respiratory rate/Diaphragm thickening fraction			
At the enrolment	1.19 [0.84–1.55]	1.09 [0.73–2.42]	0.776
1 h after the enrolment	0.69 [0.64–1.24]	1.16 [0.61–1.61]	0.315
4 h after the enrolment	0.40 [0.32–0.78]	1.21 [0.66–1.99]	0.008
12 h after the enrolment	0.33 [0.27–0.55]	1.39 [0.83–2.00]	0.008
24 h after the enrolment	0.40 [0.29–0.43]	0.75 [0.42–1.32]	0.114
48 h after the enrolment	0.42 [0.31–0.60]	0.78 [0.69–0.78]	0.033
72 h after the enrolment	0.34 [0.32–0.58]	1.07 [0.65–1.07]	0.017
96 h after the enrolment	0.34 [0.23–0.37]	0.99 [0.71–0.99]	0.036
PaO_2_/FiO_2_ ratio (mmHg)			
At the enrolment	87 [75–92]	103 [76–148]	0.36
1 h after the enrolment	197 [164–301]	140 [112–221]	0.083
4 h after the enrolment	256 [165–423]	189 [132–246]	0.20
12 h after the enrolment	245 [152–309]	133 [115–177]	0.022
24 h after the enrolment	214 [184–406]	173 [134–204]	0.154
48 h after the enrolment	256 [197–282]	158 [121–158]	0.117
72 h after the enrolment	246 [199–382]	172 [165–172]	0.517
96 h after the enrolment	261 [225–395]	162 [152–162]	0.517
PaCO_2_ (mmHg)			
At the enrolment	31 [29–49]	30 [25–39]	0.515
1 h after the enrolment	34 [30–41]	32 [24–42]	0.696
4 h after the enrolment	32 [30–37]	30 [25–44]	0.673
12 h after the enrolment	31 [30–34]	34 [28–44]	0.445
24 h after the enrolment	32 [28–36]	31 [28–35]	0.683
48 h after the enrolment	32 [29–35]	34 [32–34]	0.383
72 h after the enrolment	32 [30–45]	40 [33–40]	0.517
96 h after the enrolment	44 [28–48]	37 [35–37]	0.883
Respiratory rate, breaths per minute			
At the enrolment	38 [36–48]	38 [36–46]	0.96
1 h after the enrolment	29 [23–36]	26 [19–34]	0.51
4 h after the enrolment	22 [19–28]	30 [25–38]	0.036
12 h after the enrolment	22 [16–25]	29 [27–34]	0.020
24 h after the enrolment	21 [16–26]	26 [22–34]	0.154
48 h after the enrolment	18 [15–22]	25 [25–25]	0.012
72 h after the enrolment	18 [18–21]	21 [20, 21]	0.133
96 h after the enrolment	18 [15–20]	23 [16–23]	0.279

Results are displayed as Medians [Interquartile Range]. *P* values are calculated with Mann–U–Whitney test

*NIV* non-invasive ventilation

## Discussion

To our knowledge, this is the first study that evaluates DTF as a predictive index of NIV outcome in patients with de-novo ARF admitted to the emergency department.

Our study shows that DTF with a cut-off lower than 36.3% could predict NIV failure in hypoxemic patients, confirming previous results in other population [18, 26, 27]. In a prospective observational study including 63 patients with ARF of various origin, Di Nino et al. [18] found that a cut-off value of DTF ≥ 30% predicted extubation success regardless of the used weaning technique.

In a cohort of chronic patients ventilated in PSV through a tracheostomy tube, Ferrari et al. [26] reported that a cut-off value of DTF ≥ 36% was associated with a successful weaning after spontaneous breathing trial.

In a recent systematic review, Zambon et al. [27] evaluated 20 studies including a total of 875 critically ill patients where ultrasound was performed to detect diaphragmatic dysfunction. The authors used both diaphragmatic excursion and DTF to predict extubation success or failure during weaning and reported that the optimal cut-offs ranged from 10 to 14 mm and 30–36%, respectively.

Our study is the first to focus on hypoxemic patients during NIV treatment. However, the cut-off value of DTF that identified patients at major risk of NIV failure was similar to the cut-off value reported for critically ill patients requiring invasive mechanical ventilation [27], thus suggesting that DTF assessment is reliable to detect diaphragm dysfunction in hypoxemic patients on NIV treatment, as well.

In our study, we also assessed the reproducibility of DTF measurements. The ICC represents the proportion of total variance due to the variation between the subjects [27]. An ICC equal to 1 shows that the total variance is only due to the variation between the subjects, while an ICC equal to 0 indicates that the total variance is attributed to the variation between observers. The two operators performing ultrasound assessments in our study were appropriately trained [24] and all measurements were obtained in blind. We found overall good repeatability of DTF measurements, with ICC above the 0.75 cut-off that is usually considered as an index of good agreement among operators [28, 29]. Given the expertise of our evaluators and the overall good repeatability of DTF assessment, we don’t expect that further skilled operators could affect inter-rater agreement, as reported by other authors [15].

Considering that previous studies established a correlation between DTF and respiratory workload [15, 30–32], in a preliminary analysis we also explored the possibility that the respiratory rate/DTF ratio could be a valid predictive index of NIV failure.

In our study, the respiratory rate/DTF ratio intended to represent an ultrasound surrogate of rapid shallow breathing index [9], one of the most used and studied weaning predictors in the clinical practice [33]. We did not report the rapid shallow breathing index since it could be not accurate in patients who were spontaneously breathing. We therefore believe that another physiologic study should be performed to compare this index with the values measured by ultrasound.

We found that the cut-off value of respiratory rate/DTF ratio > 0.6 was a reliable predictor of NIV outcome in patients with de-novo ARF admitted to the emergency department.

Recently, several authors have reported the beneficial and detrimental effects of spontaneous breathing in acutely ill patients [34, 35]. The entity of inspiratory effort that is surrogated in the clinical practice by the negative swing in the esophageal pressure (*P*_*es*_) has been indicated as a possible aggravating mechanism of lung injury in patients with hypoxemic ARF [34, 35]. On the other hand, the active movements of the diaphragm may permit to recruit the dependent lung zones and maintain the end-expiratory lung volume, while the cranial shifts of the diaphragm due to the use of excessive sedation and/or neuromuscular blockade drugs may cause a significant decrease in end-expiratory lung volume [36]. Evidence for beneficial effects of spontaneous breathing has been provided only for patients with normal lungs and less severe forms of ARDS who have mild ventilatory requests [37]. In these patients, spontaneous breathing and NIV may improve gas exchange by the recruiting effect of PEEP, the improvement of hemodynamics and the avoidance of diaphragm atrophy [37].

In a recent observational study conducted on 30 patients with hypoxemic ARF who were candidate for a 24-h NIV trial, Tonelli et al. [38] found that a change in Δ*P*_*es*_ less than 10 cmH_2_0 within the first 2 h of NIV treatment was an accurate predictor of NIV outcome at 24 h, when compared to other variables. The authors also reported that in patients with moderate to severe hypoxemic ARF, the reduction in the inspiratory effort clinically translate into a significant improvement of oxygenation and a decrease of both respiratory rate and tidal volume.

Other authors [39] have also reported that the progressive development of diaphragm atrophy as well as the rapid early increases in diaphragm thickening during ventilation are associated with prolonged mechanical ventilation and an increased risk of complications.

Our study has demonstrated that a value of respiratory rate/DTF ratio > 0.6 is accurate to predict the NIV outcome in hypoxemic patients. However, we are far from understanding the mechanisms leading to worsening respiratory failure and intubation (excessive respiratory workload/inadequate unloading of the respiratory muscles versus diaphragm atrophy) since the inspiratory effort was not measured in our study population.

In hypoxemic ARF patients, NIV treatment improves respiratory discomfort [40] and reduces the need of intubation, the rate of infections and the intensive care unit mortality [41]. However, high rates of NIV failure ranging between 30 and 50% [42] are related to an increased mortality [43], most likely due to the prolonged exposure of injured lungs to high swings in *P*_*es*_ and increased tidal volumes.

In our study, the percentage of patients who failed NIV treatment and required intubation in patients with de-novo ARF was similar to the percentage reported by other authors [42], thus confirming that unsuccessful NIV is related to poor outcomes [5, 43].

### Limitations

Our study has some limitations.

First, this is a preliminary study with a small sample size that could have affected the width of sensitivity confidence interval for both operators.

Second, we did not measure inspiratory effort that is considered an early accurate predictor of NIV outcome [38].

Third, we could only evaluate the right hemi-diaphragm due to the presence of the liver as an appropriate ultrasound window that allowed us to obtain optimal images.

However, without diaphragm lesions or paralysis it is unlikely that the assessment of the left hemi-diaphragm might have changed the results of the present study.

## Conclusions

Both DTF and respiratory rate/DTF ratio may represent valid, feasible and noninvasive tools to predict the NIV outcome in patients with de-novo ARF.

Ultrasound monitoring of diaphragmatic function should be encouraged as an integral part of clinical practice when defining the correct timing of NIV discontinuation and/or the need of intubation.

However, future studies should be conducted on a large group of patients to address the ability of DTF and respiratory rate/DTF ratio to predict the NIV outcome.

## Data Availability

The datasets used and/or analysed during the current study are available from the corresponding author on reasonable request.

## Abbreviations

ARDS: Acute respiratory distress syndrome
ARF: Acute respiratory failure
ANOVA: Analysis of variance
BMI: Body Mass Index
DT: Diaphragmatic thickness
DTF: Diaphragmatic thickening fraction
Pes: Esophageal pressure
ICC: Intra-class correlation coefficient
NIV: Non-Invasive ventilation
SpO2: Peripheral oxygen saturation
PEEP: Positive end-expiratory pressure
PSV: Pressure support ventilation
PTPdi: Pressure-time product per breath
ROC: Receiver operating characteristic
RASS: Richmond Agitation-Sedation Scale
SAPS II: Simplified Acute Physiologic Score
